# Correlation of tuberculosis-related anemia severity with tuberculosis-induced inflammation in children: a six-year retrospective study

**DOI:** 10.1186/s13052-024-01664-3

**Published:** 2024-06-18

**Authors:** Chunjiao Han, Yulian Fang, Lili Dong, Detong Guo, Min Lei, Wei Guo, Chunquan Cai

**Affiliations:** 1 Tianjin Children’s Hospital, Children’s Hospital of Tianjin University, Tianjin Pediatric Research Institute, Tianjin Key Laboratory of Birth Defects for Prevention and Treatment, Tianjin, China; 2 Clinical School of Pediatrics, Tianjin Medical University, Tianjin, China; 3 Department of Pulmonology, Tianjin Children’s Hospital, Children’s Hospital of Tianjin University, Tianjin, China

**Keywords:** Tuberculosis, Anemia, Children, Inflammation

## Abstract

**Background:**

Anemia is a common complication of tuberculosis (TB), and there is evidence that its prevalence is higher in patients with TB. Although TB is very important in epidemiology, careful investigation of TB-related anemia in children has not been carried out systematically. This study aimed to describe the details of anemia in children with TB and its association with clinical characteristics and the severity of inflammation.

**Methods:**

In this retrospective study, we explored Hb levels in 103 children with pulmonary TB (PTB) and they were divided into anemic or non-anemic groups. Logistics regression analysis was used to study the associations between anemia and demographic characteristics. Spearman correlations analysis was performed to analyse the associations between the biochemical parameters and hemoglobin levels in blood.

**Results:**

The prevalence of anemia in children with TB was 37.9% (48.7% showed microcytic hypochromic anemia, and 5.1% showed normal cell anemia). Compared with the anemia (*n* = 39) group, the non-anemic group (*n* = 64) had longer fever duration and increased respiratory rate (*P* < 0.05). In logistic regression analysis, anemia was associated with lower levels of Alb and higher levels of WBC, CRP, LDH, and ESR (*P* < 0.05). Spearman correlations analysis showed a significant negative correlation between hemoglobin (Hb) levels and inflammatory markers. After one month of antitubercular therapy (ATT), the Hb levels of 76.9% children returned to normal.

**Conclusions:**

Anemia is common among children with TB at diagnosis. The majority of children with TB-related anemia are mild to moderate microcytic hypochromic anemia. There is a strong correlation between the severity of anemia and the inflammation induced by TB. This suggests that anemia is a biomarker of the severity of TB in clinical practice among children.

**Supplementary Information:**

The online version contains supplementary material available at 10.1186/s13052-024-01664-3.

## Background

Tuberculosis (TB) is a chronic respiratory infectious disease caused by *Mycobacterium tuberculosis* (MTB) infection. MTB is a rod-shaped, non-spore- forming aerobic bacterium that spreads through airborne droplets [1]. In addition, children are more likely to be infected with TB because of their immature immune system [2]. According to the World Health Organization’s 2022 Tuberculosis Report [3], it is estimated that there will be approximately 1.2 million (11%) new cases of TB worldwide in 2021, with approximately 200,000 deaths from childhood TB. Compared with adults, children with TB have a higher incidence of extrapulmonary forms and a more severe course in children < 2 years [2].

TB can cause many laboratory abnormalities such as anemia, increased erythrocyte sedimentation rate, low serum albumin level, hyponatremia, abnormal liver function, leukocytosis, and hypocalcemia [4]. Anemia is a common complication of TB and its prevalence ranges from 9.5–96% [5]. Most studies suggested that the inhibition of red blood cell generation and alteration of iron metabolism by inflammatory mediators were the main causes of anemia [6, 7]. It is an important risk factor for poor prognosis of TB, such as increased mortality rates and longer treatment times [8–10].

Mishra et al. found that serum CRP and ferritin were elevated in adult pulmonary tuberculosis patients compared with healthy individuals [11]. In addition, Leonardo et al. found that the increase of uric acid, CRP, and ESR in PTB patients was related to anemia, and the Hb concentration in patients with the highest ESR and CRP values was the lowest [12]. Edson et al. showed an association of anemia with increased C-reactive protein and hypoalbuminemia [13]. Although there have been some reports on adult TB-related anemia, few studies have evaluated TB-related anemia and its related factors in children. Our study aimed to describe the children with TB-related anemia and analyze its correlation with other hematological changes.

## Methods

### Patients and definitions

A total of 39 children with PTB-related anemia were admitted to the Respiratory Department of Tianjin Children’s Hospital between January 2018 and December 2023. Further, 64 children in PTB without anemia were hospitalized within the same period. The inclusion criteria were as follows: (a) children aged 6 months to 14 years; (b) children diagnosed with pulmonary tuberculosis; (c) children have never received antitubercular therapy (ATT) before. The diagnostic criteria for PTB were as follows [14, 15]: (1) Having symptoms and signs related to pulmonary tuberculosis. (2) Imaging is consistent with active pulmonary tuberculosis. (3) Tuberculin skin test (TST) and/or interferon-gamma release assays (IGRAs) positive. (4) Phlegm, induced sputum, bronchoalveolar lavage fluid, gastric juice, pleural effusion, tissue samples, etc. are positive for acid fast staining; MTB culture, molecular biology testing for MTB nucleic acid, or pathological results are positive. The diagnostic criteria and the severity criteria for anemia were shown in Table 1 [16]:

**Table 1:** Hb levels to diagnose anemia in children(g/l)

		Anemia		
Population	Non-Anemia	Mild	Moderate	Severe
Children 6–59 months of age	≥ 110	100–109	70–99	< 70
Children 5–11 years of age	≥ 115	110–114	80–109	< 80
Children 12–14 years of age	≥ 120	110–119	80–109	< 80

### Data collection

The clinical data of all children were collected as following: (1) basic information: name, age, gender, weight; (2) clinical manifestations: fever duration, illness duration, respiratory rate, weight loss(≥ 1 kg); (3) laboratory tests: routine blood tests, inflammatory markers, blood biochemistry.

### Statistical analysis

Statistical analyses were conducted in SPSS 26.0 and R4.3.1. The normal distribution data was represented by mean ± standard deviation or mean ± 95% confidence interval (CI). The independent sample t test was used for comparison. The non-normal distribution was described as median (P25, P75), which comparisons were made by the Mann–Whitney U-test. And the chi-square test was used for categorical data. *P* < 0.05 was considered statistically significant.

## Results

### Baseline characteristics

**Fig. 1:**
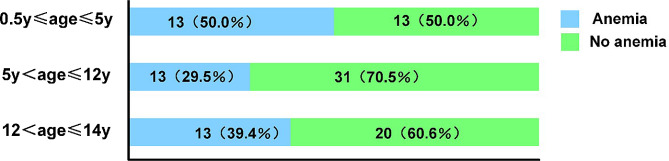
Age distribution of children with anemia

A total of 103 PTB children were included in this study. There were 39 cases (37.9%) in the anemia group (15 male; 24 female) and 64 cases (62.1%) in the non-anemia group (31 male; 33 female). The age range of anemia and non-anemia patients was 8 (1, 13) years and 11 (7, 13) years, respectively. The distribution of the number of children in different age groups was shown in Fig. 1. There was no significant difference in age, gender and weight between the two groups (*P* > 0.05), as shown in Table 2.

In terms of clinical symptoms, children in the anemia group showed longer fever duration and increased respiratory rate (*P* < 0.05). However, there was no significant difference in illness duration and weight loss between two groups (*P* > 0.05).

**Table 2:** Baseline characteristics in anemic and non-anemic children in children with PTB.

Characteristic	Anemia group(*n* = 39)	Non-Anemia group(*n* = 64)	Total (*n* = 103)	*P* value
Age(years)	8 (1, 13)	11 (7, 13)	10 (5, 13)	0.124
Gender				0.323
Male	15	31	46	
female	24	33	57	
Weight ≤ 5th percentile	2	1	3	0.660
Fever duration	7 (2, 20)	1 (0, 6)	3 (0, 11)	0.002
Illness duration	19 (7, 30)	10 (4.25, 30)	13 (5, 30)	0.122
Respiratory rate	24 (20, 28)	22 (20, 24)	22 (20, 25)	0.006
Weight loss(≥1 kg)	3	10	13	0.384

### Type and severity of anemia in children with PTB

Among 39 children with PTB-related anemia, blood tests indicated that 19 children (48.7%) had microcytic hypochromic anemia, and 2 children (5.1%) had normal cell anemia. As shown in Table 3, the severity of PTB-related anemia in most children was mild to moderate.

**Table 3:** The severity of anemia in children with PTB.

Severity	0.5y ≤ age ≤ 5y (*n* = 13)	5y< age ≤ 12y (*n* = 13)	12y< age ≤ 14y (*n* = 13)	Total (*n* = 39)
Mild	11	4	6	21
Moderate	2	7	6	15
Severe	0	2	1	3

### Biochemical indicator analysis between anemic and non-anemic TB children

**Fig. 2:**
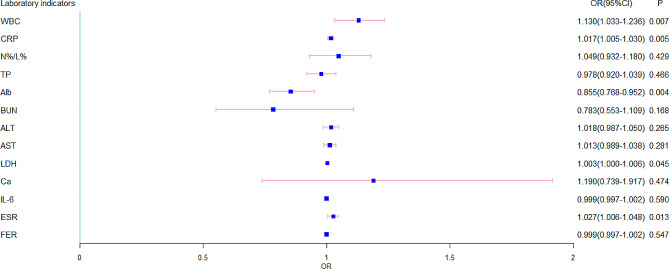
Univariate logistic regression analysis of children with anemia

In addition, this study analyzed the association between blood biochemical indicators and anemia. The results of the univariate analysis showed that WBC, CRP, Alb, LDH and ESR were significantly associated with anemia in PTB children (*P* < 0.05), as shown in Fig. 2. The increase in WBC, CRP, LDH, and ESR values was positively correlated with the occurrence of anemia, while Alb was the opposite.

### Correlation between Hb and inflammatory markers in children with tuberculosis-related anemia

**Fig. 3:**
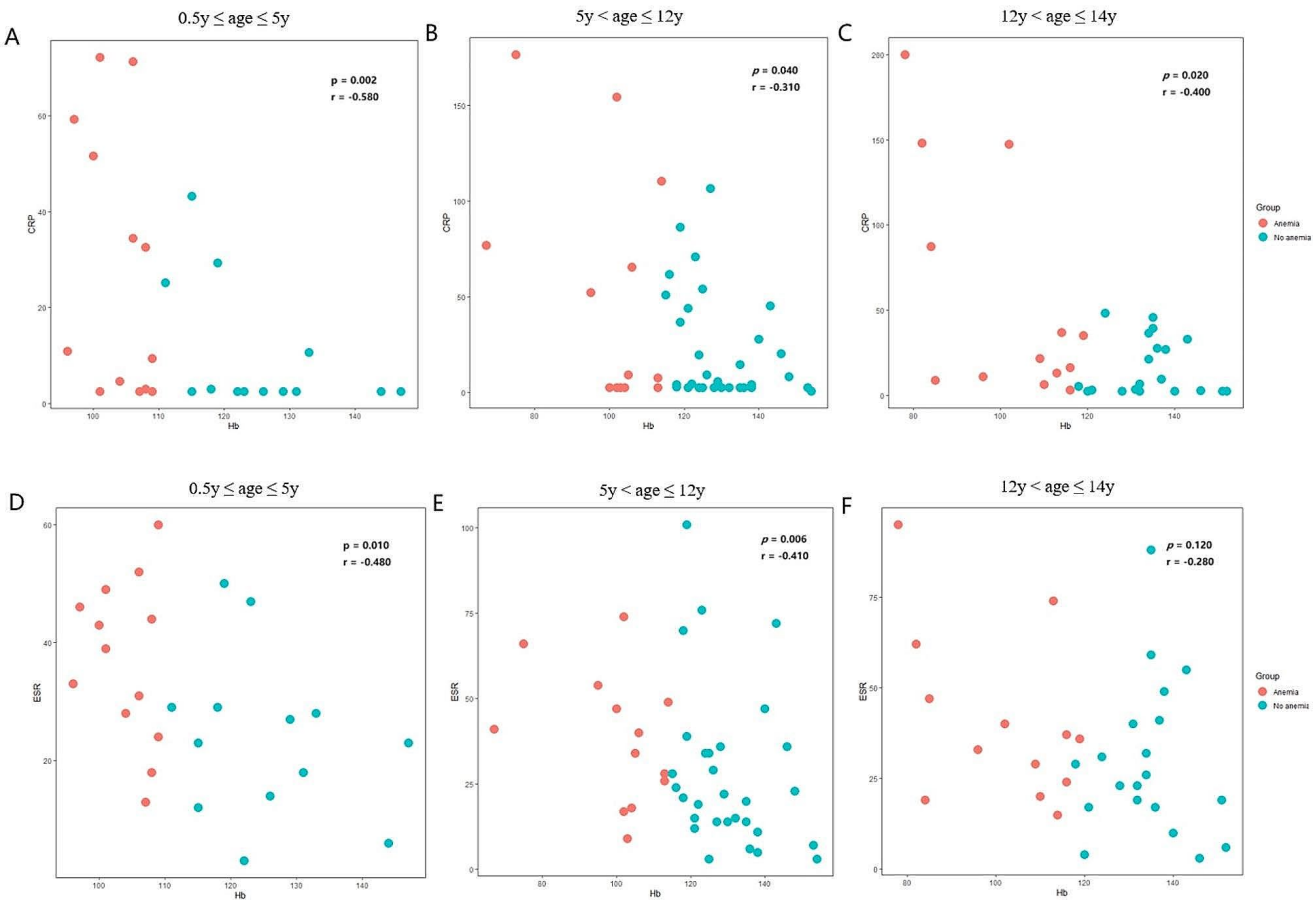
(**A**-**C**) Spearman correlation plots between CRP and Hb levels at different age groups. (**D**-**F**) Spearman correlation plots between ESR and Hb levels at different age groups

Given the results of the previous section, we analyzed that Hb values in children with PTB may be correlated with inflammatory markers. From the spearman correlation plots (Fig. 3A, B and C), it can be seen that CRP in anemic children in the three age groups is significantly higher than that of the control group, showing a negative correlation. ESR also showed a significant negative correlation between the ages of 0.5y to 5y and 5y to 12y (Fig. 3D and E), but there was no statistically significant difference between the ages of 12y to 14y (Fig. 3F). These findings clearly demonstrated that the severity of anemia was directly linked to significant alterations in the levels of inflammatory markers in children with PTB.

### Follow-up on PTB children with anemia

**Fig. 4:**
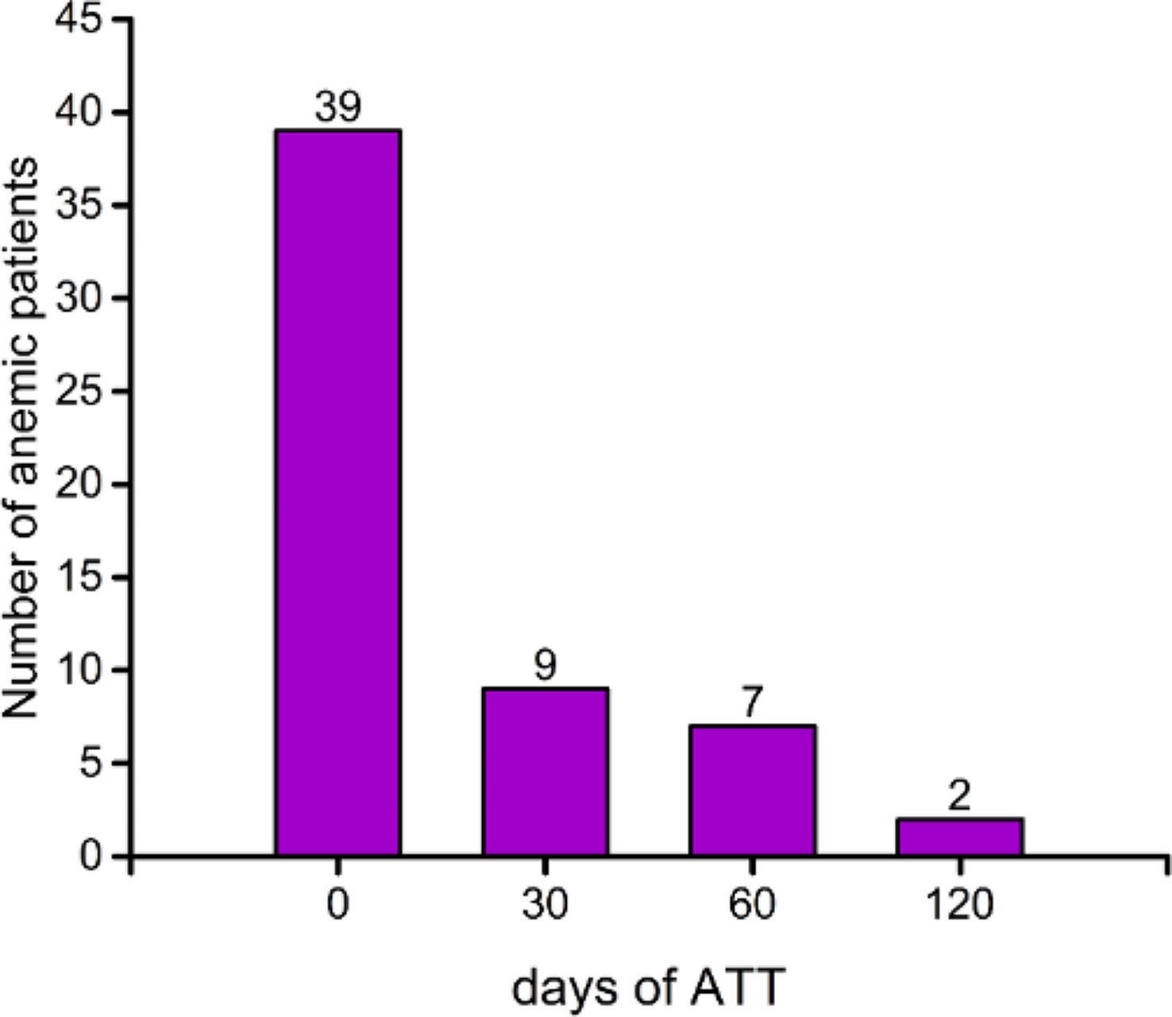
Changes in the number of anemic children with the development of ATT time

Anemic children with PTB were followed up until blood levels returned to normal after onset of ATT. The Hb levels in children with PTB-related anemia gradually return to normal with ATT, especially in the first month, with the highest number of children (76.9%) returning to normal (Fig. 4).

## Discussion

Anemia is a major health concern in children which is mainly caused by infection, malnutrition, bleeding and so on. A study found that TB patients seem to be more prone to anemia than healthy controls and those with TB in close contact [17]. TB is a risk factor for anemia, which will drive the development of anemia [18]. According to Gao et al.‘s report, the prevalence rate of TB-related anemia in children reached 58.8% [19]. Anemia can also lead to the expansion of the lung infection area, leading to lung injury and increasing the side effects of tuberculosis drugs, such as gastrointestinal distress and hepatotoxicity [8, 20]. Ashenafi et al. proposed a viewpoint that anemia is a better predictor of TB severity compared to chest X-ray results (such as PTB cavities) [21].

Although it is very important in epidemiology, careful investigation of TB-related anemia in children has not been carried out systematically. In the present study, we reported cases of TB-related anemia in children and explored its correlation with inflammation. The prevalence of anemia in children with pulmonary tuberculosis was 37.9%, which was similar to the prevalence reported by Lee et al [4]. Most children with anemia (48.7%) were microcytic hypochromic anemia, followed by hypochromic microcytic anemia (5.1%), and macrocytic anemia was not present. However, previous studies on adults have mostly reported normocytic normochromic anemia which needs further study [13, 22].

In addition to low hemoglobin levels, these TB-related anemia patients also showed elevated WBC, CRP, LDH and ESR. CRP has been found to be a useful biomarker in screening TB-HIV [23]. Many studies have observed the increase of CRP in patients with TB-related anemia [8, 24, 25]. It is speculated that CRP production may be induced by inflammatory tissue damage driven by MTB [24]. As infection progresses, chronic inflammation leads to inhibition of hemoglobin synthesis [26]. This leads to a significant inhibition of cell-mediated immune responses and the bactericidal ability of leucocytes [27, 28]. Therefore, patients with TB-related anemia may have a longer time for the proliferation and accumulation of MTB, exposing them to inflammation for a longer time [12]. This can partly explain why anemia affects the prognosis of TB.

Unlike adult studies, this study divides age into three age groups based on the diagnostic criteria for childhood anemia [16]. Our study showed a negative correlation between Hb concentrations and CRP and ESR in children with TB-related anemia. CRP and Hb values were significantly negatively correlated in three age groups. This is consistent with previous reports on anemic TB patients in adults [12]. ESR and Hb values showed a significant negative correlation in both smaller age groups, with no statistically significant difference observed in the 12–14 years age group, which may require more samples to verify. In addition, we also observed that the albumin levels in children with anemia were significantly lower than those in the non-anemia group. Hypoalbuminemia is also considered to be associated with the prognosis of TB [29, 30].

Anemia caused by TB has been proven to be restored to normal through ATT [4]. ATT leads to a decrease in peripheral blood T cell activation and related inflammatory activity by clearing MTB [12]. In our study, the incidence of anemia in children decreased by 76.9% after one month of ATT. Similarly, in the study by Leonardo and Isanaka et al., after one month of treatment with ATT, the incidence of anemia decreased by 82% and 30%, respectively [9, 12]. However, further research is needed to determine why there is such a significant difference in the incidence of anemia one month after ATT.

Our study also had some limitations. Firstly, during the follow-up treatment of children, we only had complete blood routine data and could not analysis the changes in inflammatory indicators with ATT. Secondly, it was a single-center retrospective study and the sample size was small. Further prospective studies are needed to clarify the mechanism of TB-related anemia in children.

## Conclusions

This study revealed that anemia was prevalent among children with TB at diagnosis. The majority of children with TB-related anemia were mild to moderate microcytic hypochromic anemia. There is a negative correlation between Hb values and inflammatory markers in children with TB-related anemia. Most children with TB-related anemia can recover to normal after one month of ATT treatment, suggesting a correlation between anemia and TB severity. Since blood cell count is a simple and easy detection method, it will be very effective as a biomarker of TB severity in clinical practice among children.

### Electronic supplementary material

Below is the link to the electronic supplementary material.

Supplementary Material 1

## Data Availability

All data generated or analysed during this study are included in this published article.

## Abbreviations

TB: Tuberculosis
PTB: Pulmonary tuberculosis
CI: Confidence interval
TST: Tuberculin skin test
IGRAs: interferon-gamma release assays
N%: The percentage of neutrophils
L%: The percentage of Lymphocyte
WBC: white blood cell
CRP: C-reactive protein
Alb: albumin
ESR: erythrocyte sedimentation rate
IL-6: Interleukin-6
FER: Ferritin
LDH: Lactate dehydrogenase
