# Supplementary material for: Correlation of tuberculosis-related anemia severity with tuberculosis-induced inflammation in children: a six-year retrospective study
Source: Ital J Pediatr. 2024 Jun 18;50:116. doi: 10.1186/s13052-024-01664-3 (PMC11184725; doi:10.1186/s13052-024-01664-3)
Supplement: Supplementary file 1 — Supplementary Material 1 [file 13052_2024_1664_MOESM1_ESM.docx]

Dear Reviewers,

Thank you for your comments concerning our manuscript entitled “A strong correlation between the severity of tuberculosis-related anemia and the inflammation induced by tuberculosis in children: a six-year retrospective study”. We have studied comments carefully again and have made corrections which we hope meet with approval. Revised portions are marked in red in the paper. If there are any modifications that are not in place, please kindly point them out. We would greatly appreciate it if you can kindly point out the modifications that are not in place. The main corrections in the paper and the response to your comments are as flowing:

**Reviewer #1:**

**1. Introduction: The initial section of the manuscript is notably brief and requires substantial revisions and additional elucidation to effectively engage the reader. To address this, we will extend the introduction by providing a more comprehensive overview of the study's background, significance, and objectives. This revision aims to establish a solid foundation for the reader, ensuring a clearer understanding of the research context.**

Response: Thanks for your constructive suggestion. We are very grateful for your valuable suggestions and have added more details in TB and its related anemia to lay the background for introducing the object of this study, while emphasizing the importance of this study. Please refer to the revised manuscript for details.

**2.** **Discussion: In the discussion section, it is imperative to delve deeper into the rationale behind the study's findings, offering a more detailed analysis and providing a nuanced understanding for the reader. We will augment this section by elucidating the methodology employed, addressing potential limitations, and contextualizing the results within existing literature. By doing so, we aim to enrich the reader's comprehension of the study's outcomes and facilitate a more insightful interpretation of the research findings.**

Response: Thanks for your helpful suggestion. We appreciate your valuable comments and have further analyzed the mechanism of anemia caused by TB. We have provided a more in-depth explanation of the methods and results in this study, making the problems addressed in this study at a glance. At the same time, we also proposed a hypothesis about the principle of anemia affecting the prognosis of TB. Please refer to the revised manuscript for details.

**Reviewer #2: Dear author**

**Your manuscript is interesting; I have a few comments.**

**1. The title is long: I suggest the following title: (Correlation of Tuberculosis-Related Anemia Severity with Tuberculosis-induced Inflammation in Children: A Six-Year Retrospective Study."**

Response: Thanks for your constructive comment. We have modified the title in the revised manuscript.

**2. The introduction (background) is very short and fails to review the problem despite the mass of papers on the subject.**

Response: Thanks for your helpful suggestion. We have further added the report on anemia and TB inflammation degree on the basis of the previous modification. Please refer to the revised manuscript for details.

**3. It is not clear whether you use a known international classification of anemia as mild, moderate, or severe. Please verify.**

Response: Thank you for pointing out this issue. We used a known international classification for the diagnosis and severity classification of anemia. We have updated the previous reference to WHO link in our revised manuscript.

[WHO, Haemoglobin concentrations for the diagnosis of anaemia and assessment of severity; World Health Organization; Available from: https://www.who.int/publications/i/item/WHO-NMH-NHD-MNM-11.1.pdf; Access date; March 15, 2024.]

**4. There seems to be a typographical error in the age ranges for moderate and severe anemia. For instance, the range for moderate anemia in the age group 12 years to 15 years is stated as "80 ≤ Hb ≤ 109g/L", which overlaps with the range for mild anemia. Please verify.**

Response: Thanks for your kind comment. In order to have a clearer reading, we present the diagnostic criteria and severity classification of anemia in the form of a table. Table 1 is shown in our revised manuscript.

| Table 1. Hb levels to diagnose anemia in children(g/l) | | | | |
| --- | --- | --- | --- | --- |
|  | | Anemia | | |
| Population | Non-Anemia | Mild | Moderate | Severe |
| Children 6-59 months of age | ≥110 | 100-109 | 70-99 | <70 |
| Children 5-11 years of age | ≥115 | 110-114 | 80-109 | <80 |
| Children 12-14 years of age | ≥120 | 110-119 | 80-109 | <80 |

**5. It would be better for the conclusions to include the final conclusion of the study without saying ( this study provides a detailed description of the.**

Response: Thanks for your helpful suggestion. We have removed explanatory statements and further refined our research findings and made revisions in the revised manuscript.

**Conclusions**

This study revealed that anemia was prevalent among children with TB at diagnosis. The majority of children with TB-related anemia were mild to moderate microcytic hypochromic anemia. There is a negative correlation between Hb values and inflammatory markers in children with TB-related anemia. Most children with TB-related anemia can recover to normal after one month of ATT treatment, suggesting a correlation between anemia and TB severity. Since blood cell count is a simple and easy detection method, it will be very effective as a biomarker of TB severity in clinical practice among children.
